# Data dimensionality reduction technique for clustering problem of metabolomics data

**DOI:** 10.1016/j.heliyon.2022.e09715

**Published:** 2022-06-09

**Authors:** Rustam, Agus Yodi Gunawan, Made Tri Ari Penia Kresnowati

**Affiliations:** a Telkom University, School of Electrical Engineering, Department of Telecommunication Engineering, Jl. Telekomunikasi No.1 Dayeuh Kolot, 40257 Kabupaten Bandung, Jawa Barat, Indonesia; b Institut Teknologi Bandung, Faculty of Mathematics and Natural Sciences, Industrial and Financial Mathematics Research Group, Jl. Ganesha 10 Bandung 40132, Indonesia; c Institut Teknologi Bandung, Faculty of Industrial Technology, Food and Biomass Processing Technology Research Group, Jl. Ganesha 10 Bandung 40132, Indonesia

**Keywords:** Metabolomics, Chemometric, Metabolite data, Dimensionality reduction, Indonesian clove buds

## Abstract

In metabolomics studies, independent analyses or replicating the metabolite concentration measurements are often performed to anticipate errors. On the other hand, the size of the dataset is increasing. For clustering purposes, obtaining representative information chemically from independent analyses is needed. The objective of this study is to develop a data reduction method such that a dataset that represents chemical information is obtained. Overall a proper data reduction method would simplify the clustering of metabolite data. We propose the modified Weiszfeld algorithm (MWA) to reduce independent analyses. To obtain comprehensive results, we compare MWA with some other well-known reduction methods, including PCA, CMDS, LE, and LLE. Then reduced datasets are clustered using the fuzzy c-means (FCM) algorithm with the Tang Sun Sun (TSS) index and silhouette index as the cluster validity indices. The results show that MWA, together with PCA, present the optimal number of clusters, namely four clusters. This result aligns with the optimal number of clusters before dimensionality reduction. The present results show that MWA is robust to perform dimensionality reduction of independent analyses while maintaining chemical information on the reduced dataset. Therefore, we recommend the reliability of MWA as one of the chemometric techniques, and the present finding has enriched chemometric techniques in metabolomics studies.

## 1 Introduction

The term metabolomics was introduced about 20 years ago. Since then, metabolomics has seen a tremendous increase in analytics platforms and data analysis [2], [11], [14]. Metabolomics is a comprehensive study related to identifying and quantifying all metabolites (small molecules) in a biological system [16], [38]. A complete picture of an organism's metabolic status and biochemical processes can be obtained by analyzing metabolites in a biological sample [42].

Mass spectrometry (MS) and nuclear magnetic resonance (NMR) are two instruments in metabolomics that have been widely utilized to record the status or metabolic state of biological systems [1], [26], [34], [57]. MS comes in different versions and settings, as stand-alone instruments and in combination with chromatographic separation instruments such as gas chromatography (GC) and liquid chromatography (LC). GC-MS and LC-MS are combinations of MS with chromatographic separation instruments. Using the GC-MS instrument makes it possible to characterize natural product plant compounds with high chemical diversity [21], [53]. Likewise, detailed chromatogram profiles of biological samples can be obtained using GC-MS characterization [18], [21]. Metabolomic data in natural product plants generally consist of large amounts of metabolite, multidimensional, and noisy measurements. A multivariate analysis known as chemometric techniques is necessary to interpret metabolomics data or to obtain meaningful information from a metabolite dataset of a natural product plant. Chemometric is a sub-discipline of chemistry that utilizes mathematics, statistics, and computer science to maximize the information of the measured metabolite dataset [41].

In this research, a metabolomic study is carried out on one of the natural plantation commodities originating from Indonesia, namely the clove buds [28]. Clove buds harvested from different regions are reported to have a specific flavor that may correspond to different metabolic profiles of the clove buds. Differentiating clove buds is needed by manufacturers of cosmetics and foodstuffs that use cloves as a mixture of their products to maintain the quality, particularly the taste, of the product. The method to distinguish the types of clove buds up to present is the conventional qualitative method, namely utilizing the services of a flavorist who tastes and smells buds to identify the aroma and taste of clove buds. The development of metabolic methods will serve as an essential basis to develop an automatic instrument to distinguish different types of clove buds. However, the complexity of the clove buds metabolite dataset hinders the direct clustering of clove buds based on their metabolite compositions. The appropriate technique is needed to handle this complexity. This paper presents a preprocessing method to reduce the size of the metabolite dataset to decrease the complexity of the metabolite dataset.

The typical metabolite dataset has a wide range of metabolite concentrations, namely from $10^{-4}$ to 10. Logarithmic transformations are employed to obtain reliable numerical data. On the other hand, some metabolic have zero concentrations that the logarithmic transformations cannot be directly applied. Metabolites having zero concentration are not removed or omitted from the dataset because the zero concentration could be caused by the limitations of the tools used to detect metabolites with small concentrations (less than $10^{-4}$). However, these metabolites may function as biomarkers of a particular origin [45]. Therefore, we replaced the zero concentration metabolite with one order less than the detected concentration of the smallest metabolite. The metabolite with a zero concentration is replaced $10^{-5}$. Variations between samples may also be high, among others, due to measurement errors. Independent analyses were normally conducted to overcome this problem. Overall these describe the characteristics of the metabolite dataset. Conducting the clustering process directly on the metabolite dataset may lead to meaningless results. For example, independent analyses or replicates of a sample may result in different clusters.

This research aims to search for representative data points (data vector) from independent analyses. In the previous research [44], we have reduced independent analyses using the median. The reduction was performed by finding the median of each metabolite. However, this method is not suitable for the independent analyses carried out in the laboratory. Independent analyses in each region should be viewed as multivariate data, not univariate data, where each metabolite can be reduced using the median. So, the reduction technique of independent analyses by finding the median of each metabolite is less precise.

**Figure 1:**
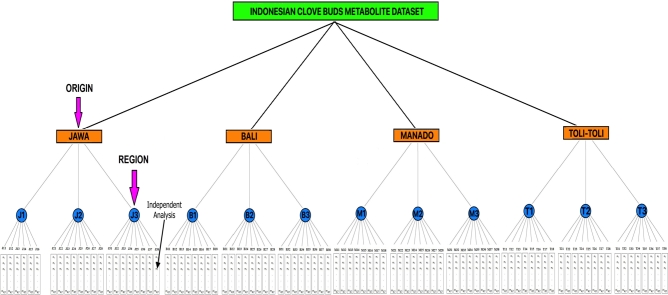
The structure of the clove bud metabolite dataset, used in this research.

The recent developments in dimensional reduction techniques on metabolomics data are many of them based on PCA technique [27], [31] and various other machine learning applications [23], [33], [35], [36]. In metabolomics studies, independent analyses are always performed to prevent errors in measuring metabolite concentrations. In this study, the independent analysis was in the metabolite data vector. A region consists of some independent analyses or vectors of metabolite data (see Fig. 1). These some independent analyses need to be reduced to a single vector of metabolite data for clustering purposes. The need to reduce some independent analyses to a single data vector avoids uninformative cluster results. The uninformative cluster results are caused by several independent analyses from the same region, leaving other independent analyses and joining clusters whose independent analyses come from other regions. The independent analysis from the same region will not differ in a cluster from other independent analyses because the independent analysis is only a repetition of experiments in a region. Therefore, a reliable data dimension reduction technique is needed to reduce some independent analyses of metabolite data vectors in each region into one metabolite data vector. In this study, we propose the modified Weiszfeld algorithm (MWA) to deal with this problem. MWA will represent some independent analyses into single data vector. MWA will search for a data vector that minimizes the total distance to all existing data vectors.

To get more comprehensive results, we compared the reduced data clustering results using our proposed MWA with several well-known dimensionality reduction methods. They were principal component analysis (PCA) [17], [24], [51], classical multidimensional scaling (CMDS) [9], [13], [56], laplacian eigenmaps (LE) [10], [48], [49], and locally linear embedding (LLE) [20], [54], [58]. The main objective of this paper is to evaluate the reliability of MWA as a data dimensionality reduction technique, specifically for metabolite data. Our focus is to compare it with several other well-known dimensionality reduction techniques. This paper does not present a comparison of clustering techniques and cluster validity indexes. So, for clustering needed, we only use the fuzzy c-means (FCM) algorithm, and for the cluster validity index, we use the Tang Sun Sun (TSS) index.

The rest of this paper is organized as follows. In Section 2, we described the real-world dataset used in this study. Furthermore, this section described the modified Weiszfeld algorithm (MWA) as a data dimensionality reduction technique, fuzzy c means (FCM) as a clustering technique, and the Tang Sun Sun (TSS) index and the silhouette index as a cluster validity indices. In Section 3, we described the results obtained and discussed them. In this section, we present a comparison of the results of clustering of reduced data using MWA with PCA, CMDS, LE, and LLE reduction techniques. Finally, in Section 4, we summarized the findings of this study.

## 2 Materials and methods

### 2.1 Dataset

This research employed a case study on the Indonesian clove buds which metabolite dataset was obtained from the research of Kresnowati et al. [28]. The dataset contained GC-MS analysis results from clove buds samples obtained from four different origins in Indonesia. Three independent clove buds samples were taken from each origin, representing different clove hubs or suppliers in that origin. We call this independent clove bud sample as region. Overall, there were twelve independent clove buds samples (region) that were extracted and analyzed to obtain the clove buds metabolite dataset. Six to eight independent analyses were performed on each of the twelve independent clove buds samples. A high number of replications were performed to anticipate errors and noise in measurements. On average, 47 metabolites were detected in each GC-MS measurement. The structure of the Indonesian clove buds metabolite dataset is shown in Fig. 1.

### 2.2 The modified Weiszfeld algorithm

**Figure 2:**
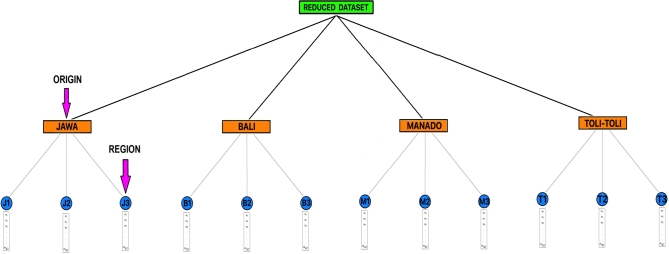
The structure of the clove bud metabolite dataset, after dimensionality reduction.

In this research, the modified Weiszfeld algorithm is proposed to reduce six or eight independent analyses (data vectors) to one data vector. It means the data matrix that was originally [47×8] or [47×6] in each region be reduced to [47×1] (see Fig. 1 and Fig. 2). This problem can be formulated mathematically, namely finding $y\in R^{d}$ which solves

(1) $$\underset{y}{\min}\left\{C(y)=\sum_{i=1}^{n}\eta_{i}\left\|y-x_{i}\right\|\right\}$$

where **y** explained the representative data point searched for each region, $x_{i}\in R^{d}$ stated independent analyses in each region, *d* represented the number of metabolites in each independent analysis, $\left\|y-x_{i}\right\|$ explained the Euclidean distance between **y** and $x_{i}$ in $R^{d}$, and $\eta_{i}$ expresses the weight associated with the Euclidean distance between $x_{i}$ and **y**. The Weiszfeld algorithm is to find a data point in $R^{d}$ that minimizes the weighted sum of Euclidean distances from the *n* given data points. Therefore, we have to find the solution of the unconstrained optimization problem in Equation (1).

The partial derivative of the objective function C(y) with respect to **y** is:

$$\frac{\partial C(y)}{\partial y}=\sum_{i=1}^{n}\eta_{i}\frac{y-x_{i}}{\left\|y-x_{i}\right\|},y\notin X$$

where $X=\{x_{1},x_{i},\cdots ,x_{n}\}\subset R^{d}$. Suppose that $y^{⁎}\notin X$ is the optimal solution of the objective function C(y), then we acquire

(2) $$\frac{\partial C(y^{⁎})}{\partial y^{⁎}}=\sum_{i=1}^{n}\eta_{i}\frac{y^{⁎}-x_{i}}{\left\|y^{⁎}-x_{i}\right\|}=0.$$

From (2), we obtain

$$y^{⁎}=\frac{\sum_{i=1}^{n}\eta_{i}\frac{x_{i}}{\left\|y^{⁎}-x_{i}\right\|}}{\sum_{i=1}^{n}\frac{\eta_{i}}{\left\|y^{⁎}-x_{i}\right\|}},$$

or $y^{⁎}=T(y^{⁎})$, where the operator $T:R^{d}\to R^{d}$ is defined by

$$T(y)=\frac{\sum_{i=1}^{n}\eta_{i}\frac{x_{i}}{\left\|y-x_{i}\right\|}}{\sum_{i=1}^{n}\frac{\eta_{i}}{\left\|y-x_{i}\right\|}}.$$

The Weiszfeld algorithm is described as follows.

**Step 1**: Initiate $y^{\left(0\right)}\notin X,\eta_{i}>0$, and ε>0. Then in the *t*-iteration, for t=0,1,2,3,⋯

**Step 2**: Calculate $T_{0}(y^{\left(t\right)})$ using

(3) $$T_{0}(y^{\left(t\right)})=\frac{\sum_{i=1}^{n}\eta_{i}\frac{x_{i}}{\left\|y^{\left(t\right)}-x_{i}\right\|}}{\sum_{i=1}^{n}\frac{\eta_{i}}{\left\|y^{\left(t\right)}-x_{i}\right\|}}.$$

**Step 3**: Update the value of **y** using

(4) $$y^{\left(t+1\right)}=\left\{ \begin{array}{ccc} T_{0}(y^{\left(t\right)}), & \mathrm{if} & y\notin X\\ x_{i}, & \mathrm{if} & y\in X \end{array}\right.$$

**Step 4**: If **y** never coincides with $x_{i}$ at each iteration, then compare $y^{\left(t\right)}$ to $y^{\left(t+1\right)}$ using $\left\|y^{\left(t+1\right)}-y^{\left(t\right)}\right\|<\varepsilon$. If true, then stop. Otherwise, set t=t+1 and return to **Step 2**. If $y=x_{i}$ occurs, stopping the iterations is performed when $y=x_{i}$ or y∈X. The Weiszfeld algorithm finds $y\in R^{d}$.

The Weiszfeld algorithms get stuck when $y=x_{i}$, it is due to division by zero in (3). So, Vardi and Zhang [52] modified the Weiszfeld algorithm to deal with the conditions $y=x_{i}$ or y∈X.

Given $y\in R^{d}$, it is convenient to write y∈X and define multiplicity at **y** as

$$\eta (y)=\left\{ \begin{array}{ccc} \eta_{k}, & \mathrm{if} & y\in X\\ 0, & \mathrm{if} & y\notin X. \end{array}\right.$$

The modification of Equation (4) for y∈X is based on the following observation. For y∉X, the vector x=T(y) in the following equation

(5) $$\overset{˜}{T}:y\to \overset{˜}{T}(y)=\frac{\sum_{i=1}^{n}\frac{\eta_{i}x_{i}}{\left\|y-x_{i}\right\|}}{\sum_{i=1}^{n}\frac{\eta_{i}}{\left\|y-x_{i}\right\|}}$$

is unique minimizer of

(6) $$f(x;y)=\sum_{i=1}^{n}\frac{\eta_{i}{\left\|x-x_{i}\right\|}^{2}}{2d_{i}(y)}.$$

So, the problem of $\mathrm{arg mi}n_{x}C(x)$ in the Weiszfeld algorithm is replaced by $\mathrm{arg mi}n_{x}f(x,y)$ in each iteration. The argument for the use of f(x;y) is

(7) $$\frac{\partial }{\partial x}f(x;y){|}_{x=y}=\frac{\partial }{\partial x}C(x){|}_{x=y},y\notin X.$$

The two minimization problems are similar in all sufficiently small neighborhoods of y,y∉X [52]. It shows that in Equation (4), if y∈X, then we should iterate with

(8) $$x^{\left(t\right)}\to \underset{x}{\mathrm{arg min}}\;f(x,x^{\left(t\right)}).$$

For this to have meaning, we need to expand the definition of *f* in Equation (6) to cover y∈X. We need to defined

$$f(x,y)=\eta (y)\left\|x-y\right\|+\sum_{x_{i}\neq y}\eta_{i}{\left\|x-x_{i}\right\|}^{2}/(2d_{i}(y))=\left\{ \begin{array}{ccc} \sum_{i=1}^{n}\eta_{i}{\left\|x-x_{i}\right\|}^{2}/(2d_{i}(y)), & \mathrm{if} & y\notin X,\\ \eta_{k}\left\|x-y\right\|+\sum_{i\neq k}\eta_{i}{\left\|x-x_{i}\right\|}^{2}/(2d_{i}(y)), & \mathrm{if} & y\in X. \end{array}\right.$$

Although C(x) is not differentiable at $x_{k}$, Equation (7) is extended for y∈X in the sense

$$\underset{x\to x_{k},x\neq x_{k}}{\lim}\left\{\frac{\partial }{\partial x}f(x,x_{k})-\frac{\partial }{\partial x}C(x)\right\}=0.$$

The modification (8) of (4) at data vectors y∈X resulting the following equation.

(9) $$y\to T(y)=\left(1-\frac{\eta (y)}{r(y)}\right)\overset{˜}{T}(y)+\min\left(1,\frac{\eta (y)}{r(y)}\right)y,$$

with the convention 0/0=0 in the computation of η(y)/r(y) where $\overset{˜}{T}$ is as in (5),

(10) $$r(y)=\left\|\overset{˜}{R}(y)\right\|,\;\;\;\overset{˜}{R}(y)=\sum_{x_{i}\neq y}\eta_{i}\frac{x_{i}-y}{\left\|x_{i}-y\right\|}.$$

For y∉X, we get $T(y)=\overset{˜}{T}(y)$, by Equation (9) with η(y)=0, as in Weiszfeld algorithm. For y∈X, T(y) is between $\overset{˜}{T}(x_{k})$ and $x_{k}$, so that by (5), T(y) is also a weighted average of **X**. Moreover, for y∉X, $\overset{˜}{R}(y)$ of Equation (10) is the negative of the gradient of C(y). It follows from Equation (5) that

(11) $$\overset{˜}{R}(y)=\left(\overset{˜}{T}(y)-y\right)\frac{\eta_{i}}{d_{i}y}.$$

Equations (11) and (10) imply that $\overset{˜}{T}(y)=(y)=T(y)$ when $r(y)=\left\|\overset{˜}{R}(y)\right\|=0$. The modified Weiszfeld algorithm is described as follows.

**Step 1**: Initiate $y^{\left(0\right)}\notin X,\eta_{i}>0$, and ε>0. Then in the *t*-iteration, for t=0,1,2,3,⋯

**Step 2**: Calculate $T_{0}(y^{\left(t\right)})$ using

(12) $$T(y^{\left(t\right)})=\frac{\sum_{y\neq x_{i}}\eta_{i}\frac{x_{i}}{\left\|y^{\left(t\right)}-x_{i}\right\|}}{\sum_{y\neq x_{i}}\frac{\eta_{i}}{\left\|y^{\left(t\right)}-x_{i}\right\|}}.$$

**Step 3**: Determine the weights

$$\eta (y)=\left\{ \begin{array}{ccc} 1, & \mathrm{if} & y\notin X\\ 0, & \mathrm{if} & y\in X \end{array}\right.$$

**Step 4**: Calculate

$$R(y^{\left(t\right)})=\sum_{y^{\left(t\right)}\neq x_{i}}\eta_{i}\frac{y^{\left(t\right)}-x_{i}}{\left\|y^{\left(t\right)}-x_{i}\right\|}$$

and

$$\psi (y^{\left(t\right)})=\min\left\{1,\frac{\eta (y^{\left(t\right)})}{\left\|R(y^{\left(t\right)})\right\|}\right\}$$

**Step 5**: Update the value of **y** using

(13) $$y^{\left(t+1\right)}=(1-\psi (y^{\left(t\right)}))T(y^{\left(t\right)})+\psi (y^{\left(t\right)})y^{\left(t\right)})$$

**Step 6**: Compare $y^{\left(t\right)}$ to $y^{\left(t+1\right)}$ using $\left\|y^{\left(t+1\right)}-y^{\left(t\right)}\right\|<\varepsilon$. If true, then stop. Otherwise, set t=t+1 and return to **Step 2**.

The condition y∉X implied $\psi (y^{\left(t\right)})=0$ and the modified Weiszfeld algorithm behave exactly as the Weiszfeld algorithm. Also, if y∉X the sum of (3) is calculated as in (12) which is only for y∉X. As for the condition y∈X is added afterwards as in (13), namely by applying the weight $\psi (y^{\left(t\right)})$ [19].

### 2.3 Fuzzy c means (FCM) algorithm

Conventional clustering means clustering the given observations as exclusive clusters. We can clearly distinguish whether an data point belongs to a cluster or not. However, such a partition is not sufficient to represent many realistic situations. Therefore, the fuzzy clustering method is offered to build clusters with uncertain boundaries. This method allows one data vector (data point) to be part of several clusters that overlap to a certain degree. In other words, the essence of fuzzy clustering is to consider the belonging status of the cluster and the extent to which objects belong to the cluster [47].

Suppose $Z=\{z_{1},z_{2},\cdots ,z_{n}\}\subset R^{d}$ is the set of *n* data points with *d* dimension to be clustered. In the case of Indonesian clove buds metabolite dataset, $z_{k}\in R^{d}(k=1,2,\cdots ,n)$ is data point that resulted from the dimensionality reduction of independent analyses in each region. Furthermore, $v_{i}\in R^{d}(i=1,2,\cdots ,c)$ is the cluster center vector of reduced dataset **Z** and c(1<c<n) in the number of clusters of the reduced dataset. The degree of membership of the data point $z_{k}$ to the cluster center $v_{i}$ can be expressed as $u_{ik}=\mu_{v_{i}}(z_{k})\in [0,1]$. The degree of membership $u_{ik}$ represents the probability of the data point $z_{k}$ to become a member of the cluster $v_{i}$.

The matrix $U=[u_{ik}]\subset R^{c\times n}$ is referred to as the fuzzy partition which filling

(14) $$u_{ik}\in [0,1],1\leq i\leq c;1\leq k\leq n,$$

(15) $$\sum_{k=1}^{n}u_{ik}>0,\forall i\in \{1,2,\cdots ,c\},$$

and

(16) $$\sum_{i=1}^{c}u_{ik}=1,\forall k\in \{1,2,\cdots ,n\}.$$

The set of all matrices satisfying (14) - (16) is denoted as $M_{fcn}$. Equation (15) guarantees that no cluster is left empty without members. The clustering process may cause some clusters to have no members. Therefore, to avoid this, (15) is needed. Equation (16) ensures that the number of degrees of membership for each data point is equal to 1. This means that each data has a degree of membership in each cluster, but with varying degrees of membership. As a consequence of (15) and (16), no cluster can contain the full membership of all data points.

One of the most widely used fuzzy clustering techniques is the fuzzy c-means algorithm [5], [8], [12], [15], [22], [29], [32]. The purpose of clustering the dataset into c fuzzy clusters is achieved by minimizing the following objective function [6].

(17) $$J_{m}(U,V;Z)=\sum_{k=1}^{n}\sum_{i=1}^{c}u_{ik}^{m}d_{ik}^{2},$$

where $V=\{v_{1},v_{2},\cdots ,v_{c}\}\subset R^{d}$ is set of cluster center, m>1 is a fuzzy parameter, and $d_{ik}^{2}$ is the Euclidean distance between $z_{k}$ with $v_{i}$. Moreover, $u_{ik}$ on the objective function $J_{m}$ shows membership degree of data vector (data point) $z_{k}$ to the cluster $v_{i}$. From the objective function $J_{m}$, we see that the FCM is the method that minimizes the weighted within-class sum of squares. Aside from assigning a data point to a cluster, membership degrees can also express how ambiguous a data point should belong to a cluster. The concept of these membership degrees is substantiated by Zadeh's definition of fuzzy set in 1965. Thus, fuzzy clustering allows solution spaces in fuzzy partitions of the dataset given. The fuzzy clustering approach with the objective function $J_{m}$ under constraints (15) dan (16) is also called probabilistic clustering, since due to the constraint (15), the membership degree $u_{ik}$ can be interpreted as the probability that data vector $z_{k}$ belongs to cluster $v_{i}$.

The optimal partition of dataset **Z** can be obtained by finding **U** and **V** which minimize the objective function $J_{m}$. The objective function $J_{m}$ reaches a local minimum when its partial derivative concerning $u_{ik}$ and $v_{i}$ is equal to zero and satisfies the constraints on (15) and (16). So we get [6]

(18) $$u_{ik}={\left(\sum_{j=1}^{c}{\left(\frac{d_{ik}^{2}}{d_{jk}^{2}}\right)}^{\frac{1}{m-1}}\right)}^{-1},1\leq i,j\leq c;1\leq k\leq n$$

and

(19) $$v_{i}=\frac{\sum_{k=1}^{n}u_{ik}^{m}z_{k}}{\sum_{k=1}^{n}u_{ik}^{m}},1\leq i\leq c.$$

Picard iteration is one of the popular algorithms for solutions (17) through (18) and (19). This type of iteration is often called alternating optimization because it only repeats through one cycle, namely $V^{\left(t-1\right)}\Rightarrow U^{\left(t\right)}\Rightarrow V^{\left(t\right)}$ and checks the stopping condition $\left\|V^{\left(t-1\right)}-V^{\left(t\right)}\right\|<\varepsilon$. This point is described in detail in [4] and [7]. Furthermore, the determination $u_{ik}$ and $v_{i}$ should be done simultaneously. However, we choose to initiate $v_{i}$ to counting $u_{ik}$ [46]. There are several advantages with initializing and terminating in $v_{i}$ in terms of convenience, convergence speed, and storage [40]. The fuzzy c-means algorithm is described as follows.

**Step 1**: Fix m>1,1<c<n, and ε>0. Initiate $v^{\left(0\right)}\in R^{d}$, $v^{\left(0\right)}$ can be selected randomly from $Z\subset R^{d}$. Then in the *t*-iteration, t=0,1,2,⋯

**Step 2**: Calculate $u_{ik}$ using

$$u_{ik}^{\left(t+1\right)}={\left(\sum_{j=1}^{c}{\left(\frac{d_{ik}^{2}}{d_{jk}^{2}}\right)}^{\frac{1}{m-1}}\right)}^{-1},1\leq i\leq c;1\leq k\leq n$$

where $d_{ik}^{2}={\left\|z_{k}-v_{i}^{\left(t\right)}\right\|}^{2}$.

**Step 3**: Update $v_{i}$ using

$$v_{i}^{\left(t+1\right)}=\frac{\sum_{k=1}^{n}{\left(u_{ik}^{\left(t+1\right)}\right)}^{m}z_{k}}{\sum_{k=1}^{n}{\left(u_{ik}^{\left(t+1\right)}\right)}^{m}},1\leq i\leq c.$$

**Step 4**: Compare $v_{i}^{\left(t\right)}$ to $v_{i}^{\left(t+1\right)}$ using $\left\|v^{\left(t+1\right)}-v^{\left(t\right)}\right\|<\varepsilon$. If true, then stop. Otherwise, set t=t+1 and return to **Step 2**.

### 2.4 Cluster validity index

In the clustering process, it is necessary to know the optimal number of clusters from a dataset. The cluster validity index was employed to determine the optimal number of clusters from the dataset.

#### 2.4.1 The Tang Sun Sun (TSS) index

The idea of this cluster validity index is to measure geometrical compactness in each cluster [25]. The Xie-Beni index [55] is widely employed to determine the number of optimal clusters. However, due to the monotone tendency to zero for c→n, the Xie-Beni index can provide a biased optimal number of clusters. The monotony nature of the Xie-Beni index has been extensively studied and discussed in various literature including [30], [39], [50]. Xie and Beni also mentioned in their paper that their cluster validity index decreased monotonically for c→n. On the other hand, the optimal number of clusters on the Xie-Beni index is indicated by the smallest value of all existing clusters 1<c<n. With the descending monotone property that converges to zero, it is possible to obtain the smallest Xie-Beni index value in the c=n−1 clusters. Therefore, to avoid the occurrence of biased cluster results, we used the Tang Sun Sun index as the cluster validity index. The Tang Sun Sun (*TSS*) index [50] does not converge to zero for c→n. The Tang Sun Sun Index is defined as follows

$$TSS(U,V;Z)=\frac{\sum_{i=1}^{c}\sum_{k=1}^{n}u_{ik}^{2}{\left\|z_{k}-v_{i}\right\|}^{2}}{\underset{1\leq i,j\leq c,i\neq j}{\min}{\left\|v_{i}-v_{j}\right\|}^{2}+\frac{1}{c}}+\frac{\frac{1}{c(c-1)}\sum_{i=1}^{c}\sum_{j=1,j\neq i}^{c}{\left\|v_{i}-v_{j}\right\|}^{2}}{\underset{1\leq i,j\leq c,i\neq j}{\min}{\left\|v_{i}-v_{j}\right\|}^{2}+\frac{1}{c}}.$$

The punishing ad hoc function on the numerator of the Tang Sun Sun index effectively eliminates the descending monotony tendency for as shown below [50].

(20) $$\lim_{c\to n}TSS(U,V;Z)=\lim_{c\to n}\frac{\sum_{i=1}^{c}\sum_{k=1}^{n}u_{ik}^{2}{\left\|z_{k}-v_{i}\right\|}^{2}}{\underset{1\leq i,j\leq c,i\neq j}{\min}{\left\|v_{i}-v_{j}\right\|}^{2}+\frac{1}{c}}+\lim_{c\to n}\frac{\frac{1}{c(c-1)}\sum_{i=1}^{c}\sum_{j=1,j\neq i}^{c}{\left\|v_{i}-v_{j}\right\|}^{2}}{\underset{1\leq i,j\leq c,i\neq j}{\min}{\left\|v_{i}-v_{j}\right\|}^{2}+\frac{1}{c}}=0+\frac{\frac{1}{n(n-1)}\sum_{i=1}^{n}\sum_{j=1}^{n}{\left\|z_{i}-z_{j}\right\|}^{2}}{\underset{i\neq j}{\min}{\left\|z_{i}-z_{j}\right\|}^{2}+\frac{1}{n}}=\frac{\sum_{i=1}^{n}\sum_{j=1,j\neq i}^{n}{\left\|z_{i}-z_{j}\right\|}^{2}}{n(n-1)\underset{i\neq j}{\min}{\left\|z_{i}-z_{j}\right\|}^{2}+(n-1)}$$

Equation (20) indicates the Tang Sun Sun index does not converge to zero for c→n. The optimal number of clusters on the Tang Sun Sun index is indicated by the smallest value of all existing clusters (1<c<n).

#### 2.4.2 The silhouette index

To obtain a more comprehensive result, we also used the silhouette index [43] to compare the TSS index as cluster validity used to determine the optimal number of clusters. In constructing the silhouette index, two things are needed. First, partition the datasets obtained using the clustering technique (we use the FCM algorithm) in this study. Second is the collection of similarities between data vectors. The similarity between data vectors is represented in the Euclidean distance between data vectors.

In the context of fuzzy clustering, the data vector $z_{k}$ is closer to the cluster center $v_{i}$ than the other data vectors, meaning that the membership degree $u_{ik}$ is greater than $u_{jk}$, namely $u_{ik}>u_{jk}$ for every *j*, where j∈{1,…,c},i≠j. Suppose that the average distance of the data vector $z_{k}$ to all data vectors in its cluster ($v_{i}$) is denoted as $a_{ik}$. Let also the minimum distance of data vector $z_{k}$ to all data vectors belonging to other clusters $v_{j},i\neq j$ is denoted as $a_{jk}$. Then, the silhouette index of the data vector $z_{k}$ is defined as [43]

$$s_{k}=\frac{a_{jk}-a_{ik}}{\max\{a_{ik},a_{jk}\}}.$$

The highest index value indicates the optimal number of clusters in the silhouette index.

## 3 Results and discussions

In the modified Weiszfeld (MWA) algorithm, weight $\eta_{i}$ is set equal to 1. It is important to note that the Weiszfeld algorithm did not analyze the weighted problem but assumed that all the weights were equal to 1. It is in line with Neumayer et al. [37] and Beck et al. [3]. Initial vector of **y** is zero vector $\left(y^{\left(0\right)}=0\right)$. It is in line with the research of Fritz et al. [19] that uses zero vector as the initial vector. In both MWA and FCM, we employed an experimental condition of $\varepsilon =10^{-5}$ and maximum number of iterations = 100. While the fuzzy parameter (*m*) in FCM, Pal and Bezdek [39] suggested the fuzzy parameter value ranging from 1.5 to 2.5. In this study, we employed the median of that values, namely m=2.

Euclid's norm is squared in clustering to tighten the clustering process. Meanwhile, using Euclid's norm in dimension reduction tends to be looser than the clustering process. We target only one data vector to represent six or eight independent analyses in each region in dimensional reduction. Meanwhile, the reduced dataset clustering process was carried out more thoroughly using the squared Euclid's norm. Reduced datasets to clusters are assigned more strictly by applying the squared Euclid's norm.

**Figure 3:**
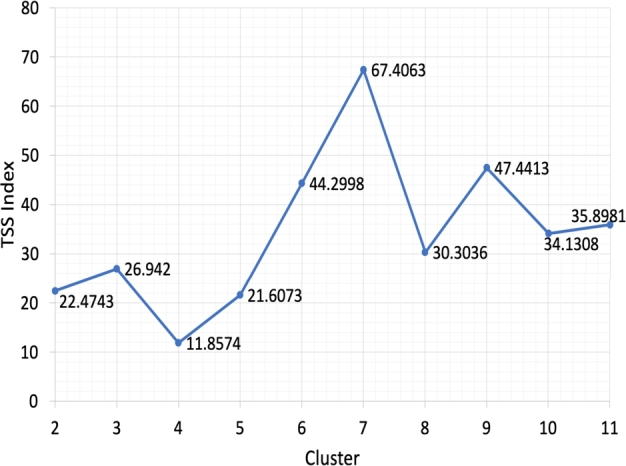
The Tang Sun Sun index values without dimensionality reduction.

**Figure 4:**
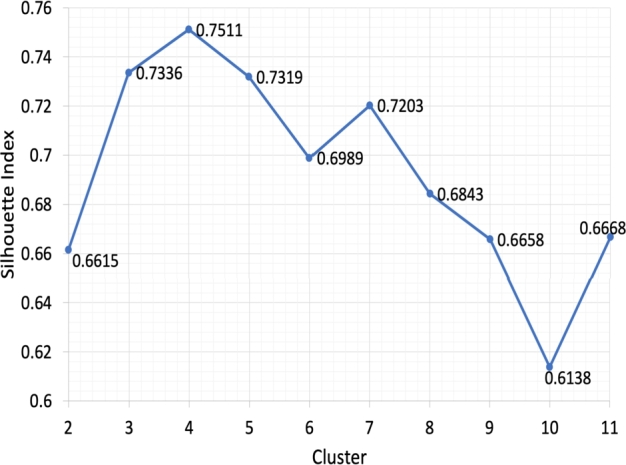
The silhouette index values without dimensionality reduction.

In this study, we first replaced the zero-concentrated metabolites with $10^{-5}$. Furthermore, the dataset is transformed using logarithmic transformation. The results of the transformation are immediately clustered without any dimensional reduction on each region. The TSS and silhouette indices values for each cluster are given in Fig. 3 and Fig. 4, respectively.

Fig. 3 shows the smallest value of the TSS index on four clusters. It means the optimal number of clusters is four clusters. Meanwhile, Fig. 4 shows the highest index value for the silhouette index, namely four clusters, which means the optimal number of clusters is four. Both cluster validity indices provide the same optimal number of clusters, namely four clusters. Details of cluster members from each cluster are shown in Table 1.

**Table 1:** Clustering result without dimensionality reduction.

Cluster	Member of Cluster
I	M11, M12, M13, M14, M15, M16, M17, M18, M21, M22, M23, M24, M25, M26, M27, M28, M31, M32, M33, M34, M35, M36, M37, M38, **T22, T33**
II	B11, B12, B13, B14, B15, B16, B17, B18, B21, B22, B23, B24, B25, B26, B27, B28, B31, B32, B33, B34, B35, B36, B37, B38
III	J11, J12, J13, J14, J15, J16, J21, J22, J23, J24, J25, J26, J27, J22, J31, J32, J33, J34, J35, J36, J37, J38
IV	T11, T12, T13, T14, T15, T16, T17, T18, T21, T23, T24, T25, T26, T27, T28, T31, T32, T34, T35, T36, T37, T38

M12 in Table 1 means the second independent analysis of the first region at the Manado origin. T35 means the fifth independent analysis of the third region at the Toli-Toli origin (see Fig. 1).

In general, Table 1 provides information that each origin of Indonesian clove buds has a unique or distinctive taste and aroma characteristics. It is based on the results of clustering, which show independent analyses from the same origin spreading in the same cluster. Each cluster consists of independent analyses from the same origin of the four existing clusters. However, Table 1 shows the independent analyses T22 and T33 are included in the first cluster that commonly contains independent analyses from Manado origin. This result provides biased information because two independent analyses (T22 and T33) from Toli-Toli origin become one cluster with independent analyses from Manado origin. We suspect that there are some errors in the measurement of metabolite concentrations in the independent analyses of T22 and T33, causing T22 and T33 to abandon other independent analyses from Toli-Toli origin and become one cluster with independent analyzes from Manado origin. Therefore, to obtain a more informative and meaningful clustering result, we propose dimensionality reduction of independent analyses in each region to become one representation data point (one data vector). Independent analyses are reduced in each region. The dataset that initially has six or eight independent analyses (data points/data vectors) in each region is reduced to one data point (see Figs. 1 and 2). It was done twelve times because, overall, there were twelve regions. Twelve data vectors resulting from dimensionality reduction are clustered using the fuzzy c-means (FCM) algorithm. The TSS and the silhouette indices are used to determine the number of optimal clusters.

Clustering is performed on a reduced dataset whose reduction uses PCA, CMDS, LE, LLE, and MWA. The obtained TSS and silhouette indices values are presented in Tables 2 and 3. The bold numbers in Table 2 show the smallest TSS index value for each dimension reduction technique. Meanwhile, the bold numbers in Table 3 show the highest silhouette index value for each dimension reduction technique. The bold numbers in Tables 2 and 3 respectively show the optimal number of clusters for each dimensionality reduction technique used.

**Table 2:** The Tang Sun Sun index values after dimensionality reduction.

Number of clusters	PCA	CMDS	LE	LLE	MWA
2	2.69	**1.48**	1.90	**2.11**	2.76
3	2.59	3.80	**1.82**	3.44	2.45
4	**1.99**	3.17	2.39	5.11	**1.87**
5	4.65	4.08	2.02	2.70	3.78
6	5.21	4.01	2.13	2.73	2.98
7	4.82	12.07	2.09	4.63	4.90
8	6.17	12.23	2.16	4.98	5.54
9	8.38	11.19	2.33	4.85	9.14
10	8.37	18.57	2.31	4.64	8.62
11	7.21	21.42	2.30	4.63	8.15

**Table 3:** The silhouette index values after dimensionality reduction.

Number of clusters	PCA	CMDS	LE	LLE	MWA
2	0.66	0.82	0.53	0.58	0.66
3	0.73	0.73	0.45	0.49	0.75
4	0.78	0.79	0.61	0.56	0.78
5	0.77	0.75	0.65	0.69	0.80
6	0.79	0.83	0.72	0.64	0.85
7	0.74	0.87	0.76	0.70	0.80
8	0.76	0.89	0.78	0.81	0.72
9	0.84	0.85	0.74	0.85	0.89
10	0.92	0.94	0.84	0.94	0.94
11	**0.98**	**0.99**	**0.89**	**0.99**	**0.98**

We will first analyze and interpret the results obtained in Table 2, using the TSS index as the cluster validity index. Based on Table 2, the optimal number of clusters obtained using PCA as a dimension reduction technique is four clusters. At the same time, the optimal number of clusters with dimension reduction using CMDS is two clusters. The optimal number of clusters using LE dimension reduction is three clusters. In comparison, the optimal number of clusters with dimension reduction using LLE is two clusters. Dimensional reduction using our proposed MWA gives the optimal number of clusters, namely four clusters. Details of cluster members from each obtained optimal number of clusters are shown in Tables 4, 5, 6, 7, and 8.

**Table 4:** Clustering result by using PCA as dimensionality reduction technique.

Cluster	Member of Cluster
I	M1, M2, M3
II	T1, T2, T3
III	B1, B2, B3
IV	J1, J2, J3

**Table 5:** Clustering result by using CMDS as dimensionality reduction technique.

Cluster	Member of Cluster
I	J2, J3, T2 B1, B2, B3 M1, M2, M3
II	J1, T1, T3

**Table 6:** Clustering result by using LE as dimensionality reduction technique.

Cluster	Member of Cluster
I	B1, B3, M1
II	J1, J2, J3 M2, T1
III	B2, M3, T2, T3

**Table 7:** Clustering result by using LLE as dimensionality reduction technique.

Cluster	Member of Cluster
I	B2, B3, T1 M1, M2, M3
II	J1, J2, J3 B1, T2, T3

**Table 8:** Clustering result by using the proposed MWA dimensionality reduction technique.

Cluster	Member of Cluster
I	M1, M2, M3
II	B1, B2, B3
III	J1, J2, J3
IV	T1, T2, T3

Table 4 shows the members of each cluster from the four optimal clusters obtained by dimension reduction using PCA. The smallest TSS index value is 1.99. It shows that the optimal number of clusters is four clusters. The results of this clustering present regions originating from the same origin, including in the same cluster. If we compare the results of the cluster before the dimension reduction in Table 1, then we find that the results of clustering with dimension reduction using PCA give the same cluster results. In general, Table 1 presents information that the independent analyses contained in each region with the same origin have the same characteristics and properties because the independent analyses are spread out in the same cluster. Likewise, after dimensional reduction using PCA, regions originating from the same origin are also in the same cluster. So, it can be concluded that PCA can perfectly reduce six or eight independent analyses in each region into one representative data vector. PCA can absorb maximum chemical information in each region without changing the chemical information in each region.

Table 5 shows the members of each cluster from the two optimal clusters obtained by dimension reduction using CMDS. The smallest TSS index value is 1.48. It means the optimal number of clusters is two. Table 5 provides information that the origin of Jawa, Bali, and Manado has the same chemical properties. Except for the region of Jawa 1 (J1) is in a different cluster, namely being one cluster with the Toli-Toli 1 (T1) and Toli-Toli 3 (T3) regions. The reduction results using CMDS provide a clustering result; the Java 1 (J1) region is separated from other regions in the origin of Jawa. Likewise, the Toli-Toli 2 (T2) region separated from other regions at the origin of Toli-Toli. It is contrary to the results shown in Table 1 that the taste and aroma of cloves from the same origin are not significantly different. So it can be concluded that dimensional reduction using CMDS cannot represent or maintain chemical information in each region as before dimensional reduction was carried out.

Table 2 shows the LE dimension reduction technique presents the smallest TSS index value of 1.82, meaning the optimal number of clusters is three. Meanwhile, LLE presents the smallest TSS index value, 2.11, which means the optimal number of clusters is two clusters. The clustering results with dimension reduction using LE and LLE presented in Tables 6, and 7 indicate that these two-dimensional reduction methods cannot maintain chemical information in each region. It is evidenced by the results of the clustering presented in Tables 6 and 7 which are mixed in one cluster of regions originating from different origins. Besides that, the results of the cluster do not reflect the distribution of the data before the dimension reduction of the independent analyses is carried out as presented in Table 2. So, LE and LLE are not good enough for dimensionality reduction of independent analysis in each region.

Furthermore, we present the results obtained by the reduction technique using MWA. Our MWA proposal presents the smallest TSS index value of 1.87, which means the optimal number of clusters is four clusters. Table 8 shows the results of data clustering with reduction of independent analyses in each region using MWA. These results indicate that the optimal number of clusters obtained in four clusters. Each cluster consists of regions from the same origin. These results align with the clustering results with reduced dimensions of independent analyses using PCA. PCA and MWA both present four optimal clusters, each cluster consisting of regions with the same origin. Our proposed MWA can consistently represent six or eight independent analyses in each region into one representative while maintaining chemical information in each region. MWA presents the results of clustering, which are in line with the results obtained in Table 1 before the dimension reduction was carried out. Based on these results, we confirm that our proposed MWA is robust for dimensionality reduction of independent analyses. Six or eight independent analyses in each region can be well represented into a single data vector while maintaining chemical information in each region.

Chemically, it can be interpreted that the data clustering of clove metabolites with dimension reduction of independent analyses using MWA indicates each clove origin has a unique chemical composition or, in other words, each clove origin has a distinctive taste and aroma. Therefore, if the production stock of a clove origin is not available, then the other available clove origin cannot be used to replace it because it has a different taste and aroma. In terms of producers who use cloves as an ingredient in their product mix, cloves from different origins will provide different product quality because each clove origin has a unique taste and aroma based on the results of this clustering.

Here, we analyze the optimal number of clusters obtained with the cluster validity index using the silhouette index. Table 3 shows the optimal number of clusters with dimension reduction techniques using PCA, CMDS, LE, LLE, and MWA are 11 clusters. It is based on the highest silhouette index value obtained for each reduction technique at the position of 11 clusters. Based on Table 9, the silhouette index does not reflect the optimal number of clusters before the independent analyses are reduced. The optimal number of clusters with the silhouette index as the cluster validity index before the reduction of independent analyses are four clusters. Meanwhile, after independent analysis reduction, each reduction technique provides an optimal number of 11 clusters with the silhouette index as the cluster validity index. The results of this clustering show that each region is in a different cluster, except for the Jawa 2 (J2) and Jawa 3 (J3) regions in the same cluster. This result means that each region has unique characteristics except for J2 and J3, which have the same characteristics. These regions come from the same origin; for example, the Manado 1 (M1), Manado 2 (M2), and Manado 3 (M3) regions come from the origin of Manado, which is still in the same area. So, there is no significant difference in climate, environmental conditions, and soil conditions. Therefore, regions of the same origin should also not be significantly different. However, this fact is different from the cluster results obtained with the silhouette index as the cluster validity index. So, we conclude that the silhouette index is not suitable for evaluating the optimal number of clusters after reducing independent analyses. The uniform optimal number of clusters, namely 11 clusters for each dimension reduction technique, also indicates the inaccuracy of the silhouette index in evaluating the optimal number of clusters after the reduction of independent analyses. Therefore, we confirm that the TSS index is more suitable because it can maintain the chemical information contained in each region before independent analysis reduction by the reduction technique using PCA and MWA that we propose.

**Table 9:** Clustering result by using the silhouette index as cluster validity index.

Cluster	Member of Cluster
I	B1
II	B3
III	J2, J3
IV	T2
V	B2
VI	T1
VII	M3
VIII	M1
IX	J1
X	T3
XI	M2

Finally, based on the results, we confirm the reliability of our proposed MWA as a chemometric technique in metabolomics studies.

**Figure 5:**
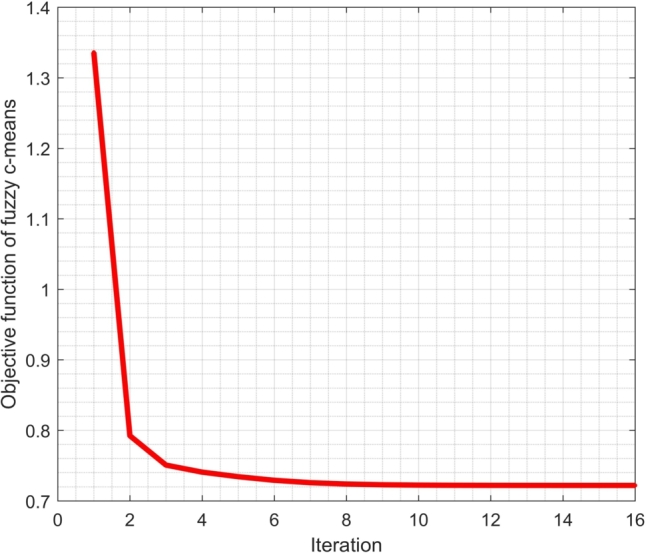
The convergence of the FCM objective function with dimension reduction using MWA.

Furthermore, the plot of the value of the objective function of the FCM algorithm for dimension reduction using MWA is shown in Fig. 5. Fig. 5 shows the convergence of the FCM objective function with dimension reduction using our proposed MWA. The value of the objective function decreases drastically from the first to the second iteration and starts to slope from the third to the eighth iteration. It appears that the objective function starts to converge to a value of 0.72 from the tenth to the sixteenth iteration. It means that the objective function has reached its minimum value since the tenth iteration. In this study, we used one of two iteration termination criteria. The first criterion is the iteration will stop when the difference in the value of the objective function in the previous and subsequent iterations is less than the specified error tolerance. In this case, the error tolerance set is $\varepsilon =10^{-5}$. If the first criterion is not met, the iteration will stop when the specified maximum iteration is reached. Here, we used a maximum number of iterations of 100. The plot of the objective function values in Fig. 5 shows that the iteration stops at the sixteenth iteration because it meets the first criterion. The objective function reaches a minimum value by obtaining four fuzzy clusters for the Indonesian clove buds metabolite dataset.

## 4 Conclusions

In this paper, we have presented the performance of the modified Weiszfeld algorithm (MWA) for dimensionality reduction of independent analyses in each region. We compared MWA with some other well-known dimensionality reduction methods to obtain more complete results, including PCA, CMDS, LE, and LLE. The results revealed that MWA, together with PCA, could provide dimensionality reduction of independent analyses in each region, consisting of six or eight independent analyses into one data point (data vector) while maintaining the chemical information of each region. The clustering results are relevant to the clustering results of the clove buds metabolite dataset before dimensionality reduction. Therefore, we recommended that MWA is reliable for dimensionality reduction of metabolite datasets consisting of independent analyses to anticipate errors in measuring metabolite concentrations. In addition, we have also presented a clove differentiation technique based on its metabolite composition, which so far has only been carried out using conventional qualitative methods utilizing the services of a taste expert (flavorist). Based on the cluster results obtained by dimensional reduction using MWA, we concluded that of the four Indonesian clove buds origins clustered, the optimal number of clusters is four clusters. It means each clove bud's origin has unique characteristics or has a distinctive taste and aroma. Finally, we recommended the reliability of MWA as one of the chemometric techniques whose use can be used more widely in metabolomics studies. This paper has enriched chemometric techniques in metabolomics studies.

## Declarations

### Author contribution statement

**Rustam**: Conceived and designed the experiments; Analyzed and interpreted the data; Contributed reagents, materials, analysis tools or data; Wrote the paper. **Agus Yodi Gunawan**: Analyzed and interpreted the data; Contributed reagents, materials, analysis tools or data; Wrote the paper. **Made Tri Ari Penia Kresnowati**: Analyzed and interpreted the data; Contributed reagents, materials, analysis tools or data; Wrote the paper.

### Declaration of interests statement

The authors declare no conflict of interest.

### Data availability statement

The data that has been used is confidential.

### Funding statement

This research did not receive any specific grant from funding agencies in the public, commercial, or not-for-profit sectors.

### Additional information

No additional information is available for this paper.
